# Autism and Intellectual Disability-Associated KIRREL3 Interacts with Neuronal Proteins MAP1B and MYO16 with Potential Roles in Neurodevelopment

**DOI:** 10.1371/journal.pone.0123106

**Published:** 2015-04-22

**Authors:** Ying F. Liu, Sarah M. Sowell, Yue Luo, Alka Chaubey, Richard S. Cameron, Hyung-Goo Kim, Anand K. Srivastava

**Affiliations:** 1 J.C. Self Research Institute of Human Genetics, Greenwood Genetic Center, Greenwood, South Carolina, United States of America; 2 Department of Genetics and Biochemistry, Clemson University, Clemson, South Carolina, United States of America; 3 Department of Medicine, Georgia Regents University, Augusta, Georgia, United States of America; 4 Department of OB/GYN, Institute of Molecular Medicine and Genetics, Georgia Regents University, Augusta, Georgia, United States of America; Emory University, UNITED STATES

## Abstract

Cell-adhesion molecules of the immunoglobulin superfamily play critical roles in brain development, as well as in maintaining synaptic plasticity, the dysfunction of which is known to cause cognitive impairment. Recently dysfunction of KIRREL3, a synaptic molecule of the immunoglobulin superfamily, has been implicated in several neurodevelopmental conditions including intellectual disability, autism spectrum disorder, and in the neurocognitive delay associated with Jacobsen syndrome. However, the molecular mechanisms of its physiological actions remain largely unknown. Using a yeast two-hybrid screen, we found that the KIRREL3 extracellular domain interacts with brain expressed proteins MAP1B and MYO16 and its intracellular domain can potentially interact with ATP1B1, UFC1, and SHMT2. The interactions were confirmed by co-immunoprecipitation and colocalization analyses of proteins expressed in human embryonic kidney cells, mouse neuronal cells, and rat primary neuronal cells. Furthermore, we show KIRREL3 colocalization with the marker for the Golgi apparatus and synaptic vesicles. Previously, we have shown that KIRREL3 interacts with the X-linked intellectual disability associated synaptic scaffolding protein CASK through its cytoplasmic domain. In addition, we found a genomic deletion encompassing *MAP1B* in one patient with intellectual disability, microcephaly and seizures and deletions encompassing *MYO16* in two unrelated patients with intellectual disability, autism and microcephaly. MAP1B has been previously implicated in synaptogenesis and is involved in the development of the actin-based membrane skeleton. MYO16 is expressed in hippocampal neurons and also indirectly affects actin cytoskeleton through its interaction with WAVE1 complex. We speculate KIRREL3 interacting proteins are potential candidates for intellectual disability and autism spectrum disorder. Moreover, our findings provide further insight into understanding the molecular mechanisms underlying the physiological action of KIRREL3 and its role in neurodevelopment.

## Introduction

**Fig 1:**
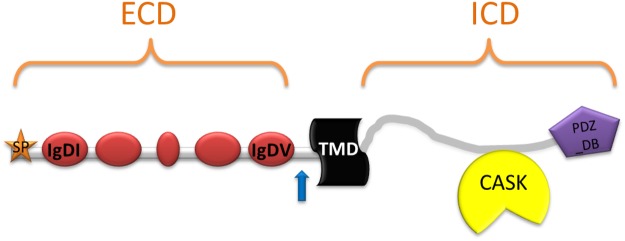
Schematic representation of the KIRREL3 domains. Five immunoglobulin domains (IgD), a signal peptide (SP) region, a transmembrane domain (TMD), and a PDZ- domain binding motif (PDZ-BD) are shown. ECD, extracellular domain; ICD, intracellular domain. The blue arrow indicates a potential cleavage site.

Intellectual disability (ID) is a genetically and clinically heterogeneous condition characterized by below-average intellectual functioning (IQ<70) in conjunction with significant limitations in adaptive functioning. We and others have recently identified a potential role for human KIRREL3, a mammalian homologue of the gene *Kirre* (kin of irregular chiasm C-roughest) of *Drosophila melanogaster*, in neurodevelopment [1–3]. The *KIRREL3* gene, located at 11q24.2, encodes a synaptic cell-adhesion molecule of the immunoglobulin (Ig) superfamily. KIRREL3 contains five Ig like domains in its extracellular portion and a PDZ domain-binding motif in its cytoplasmic portion (Fig 1).

Defects of *KIRREL3* has been linked to several neurological and cognitive disorders including ID, neurocognitive delay associated with Jacobsen syndrome, and autism spectrum disorder (ASD) [1–3]. The gene was found to be physically disrupted by a balanced t(11;16) translocation in a patient with ID [1]. In a second patient with a neurodevelopmental disorder and a t(X;11) translocation, the breakpoint, located 39 kb upstream of the *KIRREL3* coding region, altered both mRNA and protein levels [3]. An interstitial deletion of 11q-implicating the *KIRREL3* gene in the neurocognitive delay observed in Jacobsen syndrome has recently been reported [2]. Based on the patient’s history of neurocognitive delay and autism spectrum disorder, the authors concluded that the gene was a candidate for social and expressive language delay. In addition, missense alterations in *KIRREL3* have been identified in patients with mild to severe ID [1]. Additional studies further suggested *KIRREL3* as an intriguing candidate for autism [4–5] and a potential risk gene for Alzheimer's disease [6].

Previously, we detected expression of the *KIRREL3* gene in human fetal and adult brain and found that the encoded protein is located on the cell membrane and in a distinct area in the cytoplasm. We further showed that the intracellular domain of KIRREL3 interacts with the synaptic scaffolding protein, calmodulin-associated serine/threonin kinase (CASK), an X-linked ID protein [1]. A role for murine Kirrel3 in synaptogenesis was also suggested based on its temporal and spatial expression in developing and adult mouse brain and its interaction with Cask [7]. We reasoned that the identification of brain-expressed proteins whose functions are either dependent on or associated with KIRREL3 protein may be relevant in identifying potential molecular mechanisms and pathways underlying ID. Thus we hypothesized that KIRREL3 likely binds other synaptic protein(s) to modulate its physiological action(s).

In the present study, using the yeast two-hybrid (Y2H) screening system, we identified brain expressed proteins that interact with the KIRREL3-ECD and KIRREL3-ICD. KIRREL3-ECD physically associated with MAP1BLC1 and MYO16. In addition to the previously identified interaction with CASK, KIRREL3-ICD potentially interacts with ATP1B1, UFC1, and SHMT2. All the interactions were confirmed by co-immunoprecipitation (Co-IP) and colocalization analyses in human embryonic kidney cells (HEK293H) and various neuronal cells. Furthermore, we show KIRREL3 colocalization with the Golgi apparatus and synaptic vesicles. Several of the identified interacting partners of KIRREL3, including MAP1B and MYO16, have previously been linked to neurological and cognitive disorders. Our studies provide additional information for the understanding of KIRREL3 physiological functions in neurodevelopment.

## Results

### Identification of brain expressed proteins that interact with KIRREL3

**Fig 2:**
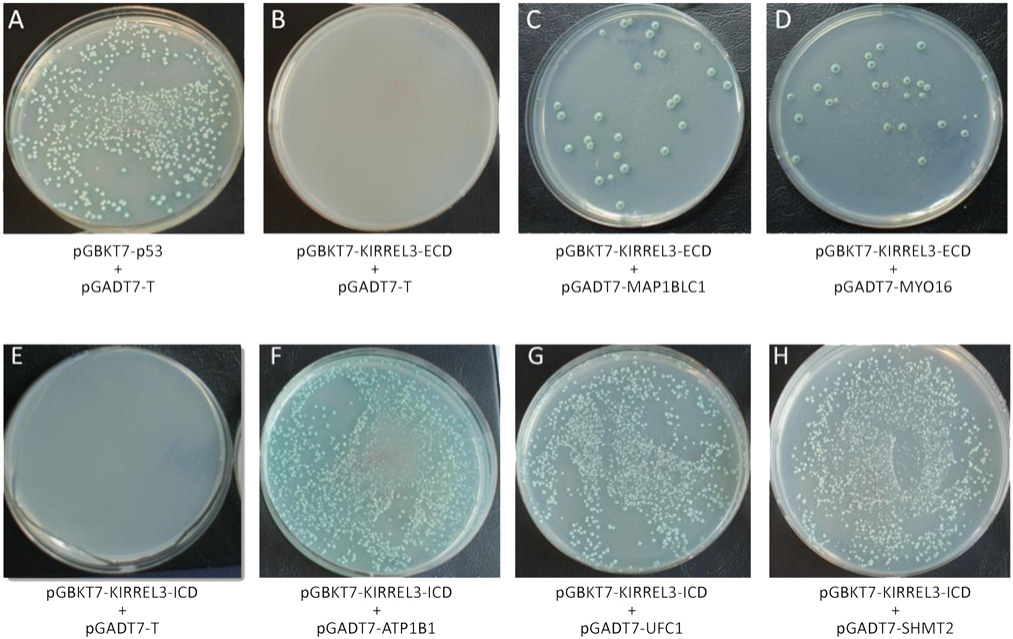
Individual yeast clones identified as potential interacting partners of KIRREL3-ECD and KIRREL3-ICD. (A) Positive control: yeast mating of [pGBKT7-p53] in AH109 and [pGADT7-T] in Y187. (B) Negative control: yeast mating of [pGBKT7- KIRREL3- ECD] in AH109 and [pGADT7-T] in Y187. (C) Yeast mating of [pGBKT7-KIRREL3-ECD] in AH109 and [pGADT7-MAP1BLC1] in Y187. (D) Yeast mating of [pGBKT7-KIRREL3 ECD] in AH109 and [pGADT7-MYO16] in Y187. (E) Negative control: yeast mating of [pGBKT7- KIRREL3-ICD] in AH109 and [pGADT7-T] in Y187. (F) Yeast mating of [pGBKT7-KIRREL3-ICD] in AH109 and [pGADT7-ATP1B1] in Y187. (G) Yeast mating of [pGBKT7-KIRREL3-ICD] in AH109 and [pGADT7-UFC1] in Y187. (H) Yeast mating of [pGBKT7-KIRREL3-ICD] in AH109 and [pGADT7-SHMT2] in Y187. Yeast mating was performed and cells were grown on—AHLT X-α-gal plates. Only clones with interacting proteins grow on—AHLT X-α-gal media and turn blue.

To gain an understanding of the physiological role of KIRREL3 in neurodevelopment, we sought to identify proteins that interact with KIRREL3. Thus, to identify brain expressed proteins that interact with the ECD of KIRREL3, we performed a Y2H screening using the Match & Plate Human Fetal Brain cDNA library (Clontech) in a prey vector (pGADT7) and KIRREL3-ECD (amino acids 1–517) in a bait vector (pGBKT7). Among ~1.4 x 10^7^ independent yeast transformants screened, several independent positive clones were identified under stringent nutritional conditions of growth (-Ade, -His, -Leu, -Trp, X-α-galactosidase). These positive prey plasmids were isolated, sequenced and analyzed. Six KIRREL3-ECD interacting positive partial cDNA clones encoded overlapping regions of the microtubule associated protein light chain (MAP1BLC1), and one positive partial cDNA clone encoded an unconventional myosin protein (MYO16). In a similar Y2H screen using the ICD of KIRREL3 (amino acids 545–766) as bait, eight partial cDNA clones encoding overlapping regions of the ATPase, Na^+^/K^+^ transporting, beta 1 polypeptide (ATP1B1), four cDNA clones encoding overlapping regions of the ubiquitin-fold modifier conjugating enzyme 1 (UFC1), and four cDNA clones encoding a full-length serine hydroxymethyltransferase 2 (SHMT2) were identified as potential KIRREL3-ICD interacting proteins. The specificities of the interactions were individually confirmed using the empty pGBKT7 vector, a negative control cDNA in pGBKT7, or a negative control in pGADT7 (pGADT7-T), and one-to-one yeast mating assays (Fig 2 and data not shown).

### Interaction of KIRREL3 with MAP1BLC1, MYO16, ATP1B1, UFC1, and SHMT2 in mammalian cells

**Fig 3:**
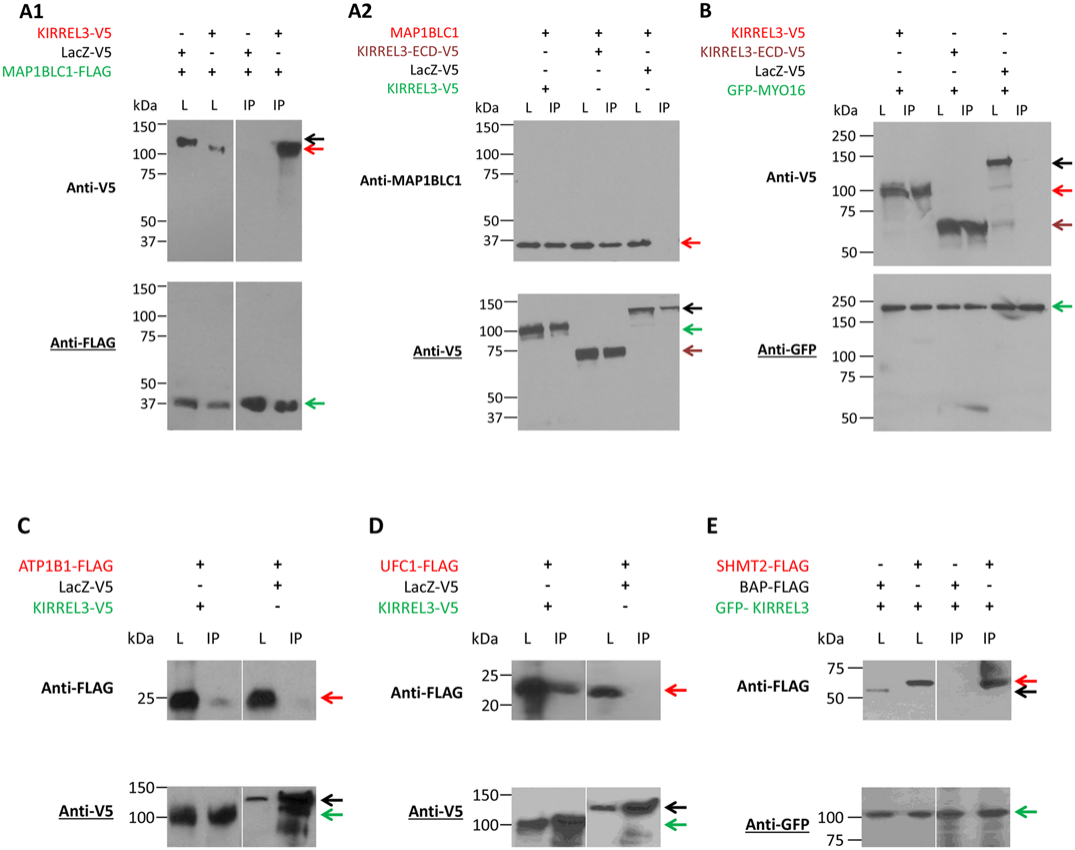
Western blot analysis of the Co-IP of KIRREL3-V5 with MAP1BLC1-FLAG (A1) and endogenous MAP1BLC1 (A2); KIRREL3-V5 with GFP-MYO16 (B), ATP1B1-FLAG (C), UFC1-FLAG (D), and GFP-KIRREL3 with SHMT2-FLAG (E). Lysates from HEK293H cells overexpressing the indicated expression constructs were incubated with anti-FLAG antibody (A1), anti-V5 antibody (A2, C, and D), and anti-GFP antibody (B, E), and precipitated with magnetic beads. Lysates from N2a cells overexpressing KIRREL3-V5 expression construct was incubated with anti-V5 antibody, and precipitated with magnetic beads (A2). Immunoprecipitates (lane IP, Co-IP) were analyzed by western blotting as indicated with anti-V5, anti-MAP1BLC1, anti-FLAG, and anti-GFP antibodies. Expression of all proteins was also analyzed in total lysates (lane L). MAP1BLC1-FLAG (green arrow) immobilizes KIRREL3-V5 (A1, lane IP, red arrow), but not LacZ-V5 (black arrow). KIRREL3-V5 (green arrow) and KIRREL3-ECD-V5 (brown arrow), but not LacZ-V5 (black arrow) immobilize endogenous MAP1BLC1 (A2, lane IP, red arrow). GFP-MYO16 (green arrow) immobilizes KIRREL3-V5 (B, lane IP, red arrow), KIRREL3-ECD-V5 (B, lane IP, brown arrow) but not LacZ-V5 (B, lane IP, black arrow). (C) KIRREL3-V5 (green arrow) but not LacZ-V5 (black arrow) immobilizes ATP1B1-FLAG (red arrow). (D) KIRREL3-V5 (green arrow) but not LacZ-V5 (black arrow) immobilizes UFC1-FLAG (red arrow). (E) GFP-KIRREL3 (green arrow) immobilizes SHMT2-FLAG (red arrow) but not BAP-FLAG (black arrow).

To confirm the interaction of full-length KIRREL3 with MAP1BLC1, MYO16, ATP1B1, UFC1, and SHMT2, we performed Co-IP analyses in HEK293H cells. The cells were co-transfected with KIRREL3-V5 and with either MAP1BLC1-FLAG or GFP- MYO16, or ATP1B1-FLAG, or UFC1-FLAG mammalian expression constructs (Fig 3A–3D), or transfected with GFP-KIRREL3 and with SHMT2-FLAG mammalian expression constructs (Fig 3E). We detected KIRREL3 in immunoprecipitates of MAP1BLC1 (Fig 3A1), and MYO16 (Fig 3B). Similarly we detected ATP1B1, UFC1, and SHMT2 in immunoprecipitates of KIRREL3 (Fig 3C–3E). Subsequently, we also confirmed interaction of the endogenous MAP1BLC1 with KIRREL3-V5 via a MAP1BLC1-specific antibody in Neuro-2a (N2a) cells (Fig 3A2). The interactions were also confirmed individually with the KIRREL3-ECD or ICD with their respective interacting partners (Fig 3A2 and 3B and data not shown). Table 1 summarizes descriptions of all KIRREL3 interacting proteins.

**Table 1:** Summary of KIRREL3 interacting proteins.

Interacting region	Protein ID	Description	Gene location	MIM	Function
**KIRREL3-ECD**	MAP1BLC1	Microtubule-associated protein 1B light chain 1	5q13	157129	Involved in the development of actin-based membrane cytoskeleton
	MYO16	Myosin XVI	13q33.3	615479	Indirectly affects actin cytoskeleton through its interaction with WAVE1 complex
**KIRREL3-ICD**	CASK	Calcium/calmodulin-dependent serine protein kinase (MAGUK family)	Xp11.4	300172	Localizes to synaptic active zones and binds to—neurexin and calcium channels
	ATP1B1	ATPase, Na^+^/K^+^ transporting, beta 1 polypeptide	1q24	182330	Involved in cell adhesion and establishing epithelial cell polarity
	SHMT2	Serine hydroxymethyltransferase 2 (mitochondrial)	12q12-q14	138450	Contributes to the *de novo* mitochondrial thymidylate biosynthesis pathway. Required to prevent uracil accumulation in mtDNA. Interconversion of serine and glycine. Associates with mitochondrial DNA
	UFC1	Ubiquitin-fold modifier conjugating enzyme 1	1q23.2	610554	E2-like enzyme which forms an intermediate with UFM1 via a thioester linkage.

### Colocalization of KIRREL3 with MAP1BLC1, MYO16, ATP1B1, UFC1, and SHMT2 in neuronal cells

**Fig 4:**
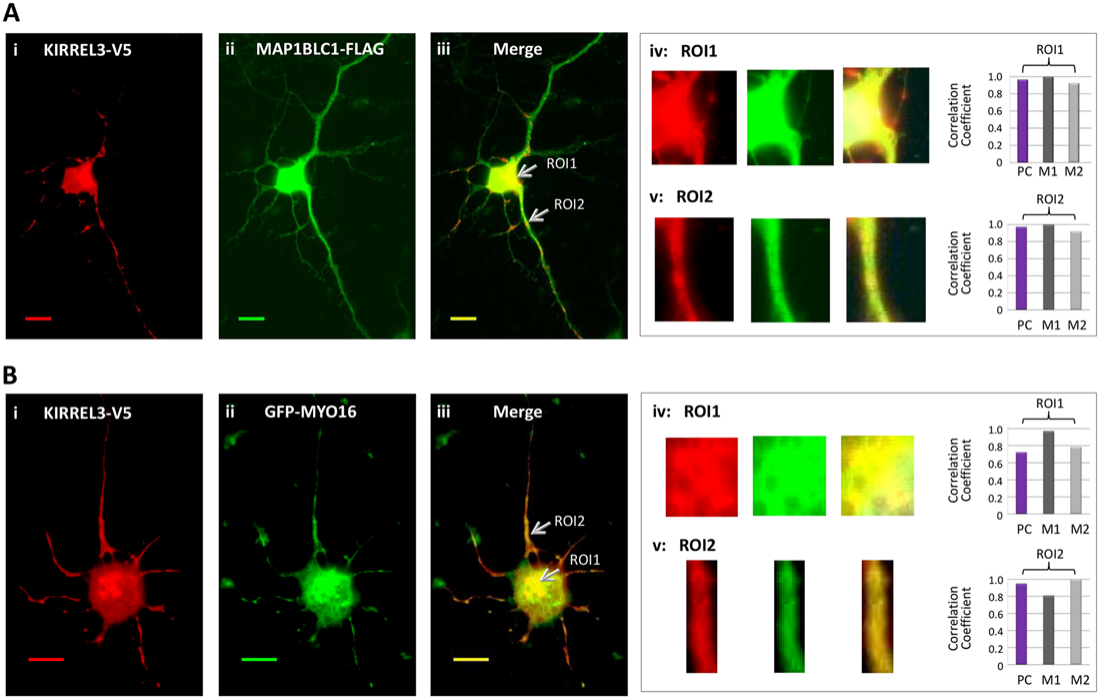
(A) KIRREL3-V5 (red, i) and MAP1BLC1-FLAG (green, ii) co-overexpressed in rat PNCs. (B) KIRREL3-V5 (red, i) and GFP-MYO16 (green, ii) co-overexpressed in rat PNCs. The overlapping signals of the two proteins appear as yellow/orange (Aiii, Biii) within the region of cytoplasm and in neurite processes (arrows). Enlarged overlay images and individual red and green channels for each ROIs (see Materials and Methods) are shown (A-B, iv and v). The degree of colocalization between the red and green signals was statistically analyzed and expressed with Pearson’s correlation coefficient (PC) and Mander’s colocalization coefficients M1 and M2 (A-B, iv and v). M1 represents the fraction of KIRREL3-V5 (red signal) overlapped with MAP1BLC1-FLAG or GFP-MYO16 (green signal). M2 represents the fraction of MAP1BLC1-FLAG or GFP-MYO16 (green signal) overlapped with the KIRREL3-V5 (red signal). All calculations for Pearson’s and Mander’s coefficients were performed by the ImageJ version 1.45s visualization software with JACoP plugin. Bar, 20μm.

To determine whether KIRREL3-ECD interacting proteins MAP1BLC1 and MYO16 colocalize with KIRREL3, immunofluorescence microscopy analysis was performed in three different cell lines, HEK293H cells, rat primary neuronal cells (PNC), and N2a cells. The distribution of KIRREL3-V5 (C-terminal tag) and GFP-tagged KIRREL3 (N-terminal tag) in PNC cells were quite similar suggesting that tagging of KIRREL3 did not influence its subcellular localization (data not shown). We cotransfected the cells with KIRREL3-V5 and MAP1BLC1-FLAG constructs (Fig 4A) or KIRREL3-V5 and GFP-MYO16 constructs (Fig 4B). Immunostaining revealed the potential colocalization of KIRREL3 with MAP1BLC1 and MYO16 (Fig 4 and data not shown). The colocalization appears to be partial as the overlapping (yellow/orange) signals were noted distinctly in selected areas in the cytoplasm (Fig 4Aiii and 4Biii), as well as in punctate structures along the entire length of neurite processes (Fig 4Aiii and 4Biii). No overlapping signals were detected using KIRREL3-V5 and GFP-empty vector without MYO16 or KIRREL3-V5 and Bacterial alkaline phosphatase (BAP)-FLAG negative control (data not shown). The quantitative evaluation of colocalization between KIRREL3-V5 and MAP1BLC1-FLAG and between KIRREL3-V5 and GFP-MYO16 was performed on region(s) of interest (ROI) (see Materials and Methods). Pearson’s and Mander’s coefficients for each regions of interest showed good correlation (Fig 4Aiv, 4Av, 4Biv, 4Bv, and Table 2). Together, both visual inspection, as well as quantitative colocalization analyses of the images confirmed the colocalization of KIRREL3 with MAP1BLC1 and with MYO16. We further confirmed the colocalization of KIRREL3-ECD with MAPL1BLC1 and MYO16 in all the three cell lines (data not shown).

**Table 2:** Quantitative colocalization analysis of immunofluorescence microscopy experiments.

Experiment Purpose	Figure ID	Red Signal	Green Signal	PC^a^	M1^b^	M2^c^
Confirm colocalization between KIRREL3 and potential interacting proteins	Fig 4Aiv_ROI1	KIRREL3-V5	MAP1BLC1-FLAG	0.965	1.000	0.923
	Fig 4Av_ROI2	KIRREL3-V5	MAP1BLC1-FLAG	0.971	1.000	0.918
	Fig 4Biv_ROI1	KIRREL3-V5	GFP-MYO16	0.720	0.971	0.784
	Fig 4Bv_ROI2	KIRREL3-V5	GFP-MYO16	0.947	0.810	1.000
	Fig 5Aiv	KIRREL3-V5	ATP1B1-FLAG	0.808	0.989	0.816
	Fig 5Biv	KIRREL3-V5	UFC1-FLAG	0.788	0.969	1.000
	Fig 5Civ	KIRREL3-V5	SHMT2-FLAG	0.843	0.923	1.000
Investigate Golgi localization of KIRREL3	Fig 6E	Golgi	GFP-KIRREL3	0.819	0.955	0.983
Investigate synaptic vesicle localization of KIRREL3	Fig 7D	KIRREL3-V5	Synaptophysin	0.917	0.940	0.864

^a^. Pearson’s correlation coefficient

^b^. Mander’s colocalization coefficient M1 represents the fraction of red signal overlapping with green signal.

^c^. Mander’s colocalization coefficient M2 represents the fraction of green signal overlapping with red signal.

**Fig 5:**
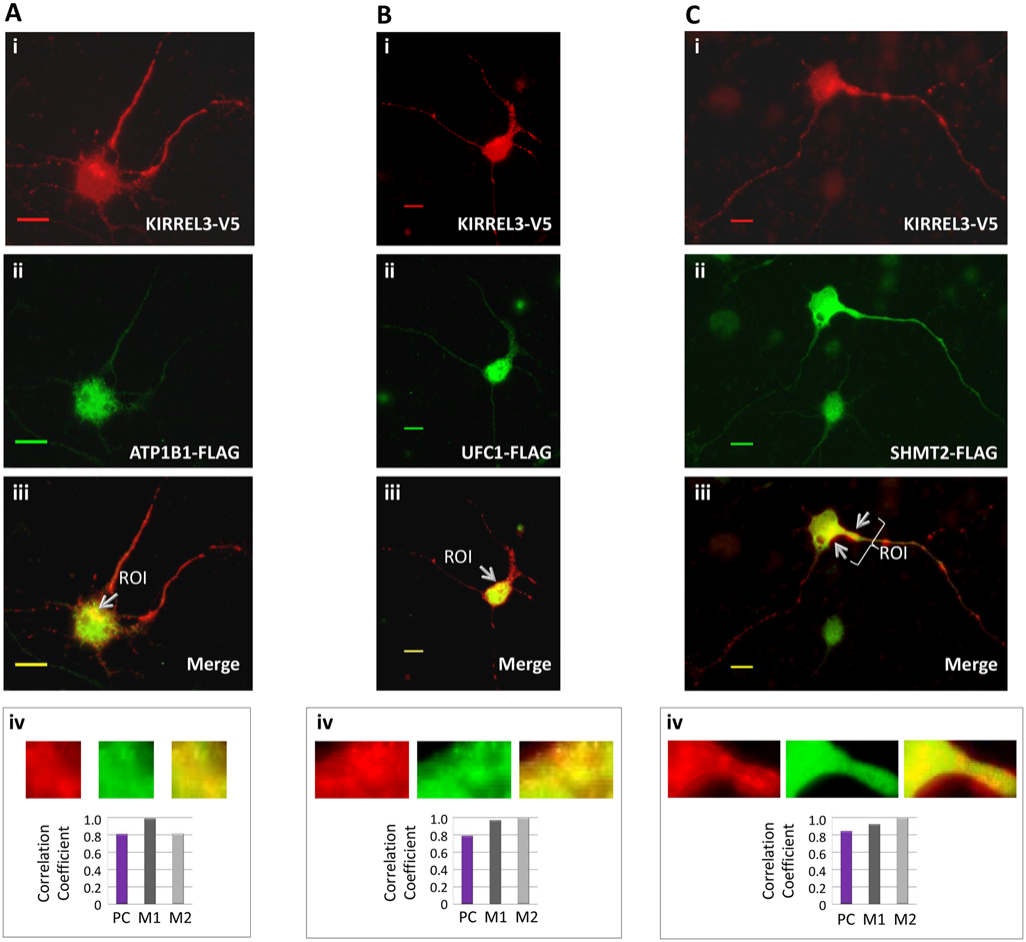
KIRREL3-V5 (red) and ATP1B1-FLAG (green) (A), KIRREL3-V5 (red) and UFC1-FLAG (green) (B), and KIRREL3-V5 (red) and SHMT2-FLAG (green) (C) co-overexpressed in rat PNCs. The overlapping signals of the two proteins appear as yellow/orange within the region of cytoplasm (A-C, iii), and in neurite processes (Ciii). Enlarged overlay images and individual red and green channels for each ROIs are shown (A-C, iv). The degree of colocalization between the red and green signals was statistically analyzed (A-C, iv) and expressed with Pearson’s correlation coefficient (PC) and Mander’s colocalization coefficients (M1 and M2). Bar, 20μm.

To determine whether ATP1B1, UFC1, and SHMT2 colocalize with KIRREL3, immunefluorescence microscopy analysis was performed in rat PNCs (Fig 5A–5C). We cotransfected the cells with KIRREL3-V5 and ATP1B1-FLAG constructs (Fig 5A), KIRREL3-V5 and UFC1-FLAG constructs (Fig 5B), and KIRREL3-V5 and SHMT2-FLAG constructs (Fig 5C). Immunostaining revealed the partial colocalization of KIRREL3 with ATP1B1, KIRREL3 with UFC1, and KIRREL3 with SHMT2 (Fig 5Aiii–5Ciii) in selected areas in the cytoplasm (Fig 5A–5C, iii), as well as in punctate structures along the entire length of neurite processes (Fig 5Ciii). Furthermore, no overlapping signals were detected using LacZ-V5 and BAP-FLAG negative controls (data not shown). We quantitatively evaluated the colocalization. Both Pearson’s and Mander’s coefficients calculated high scores for all the selected ROIs (Fig 5A–5C and iv, and Table 2), which suggested the colocalization.

### KIRREL3 colocalizes with the Golgi complex and synaptic vesicles

**Fig 6:**
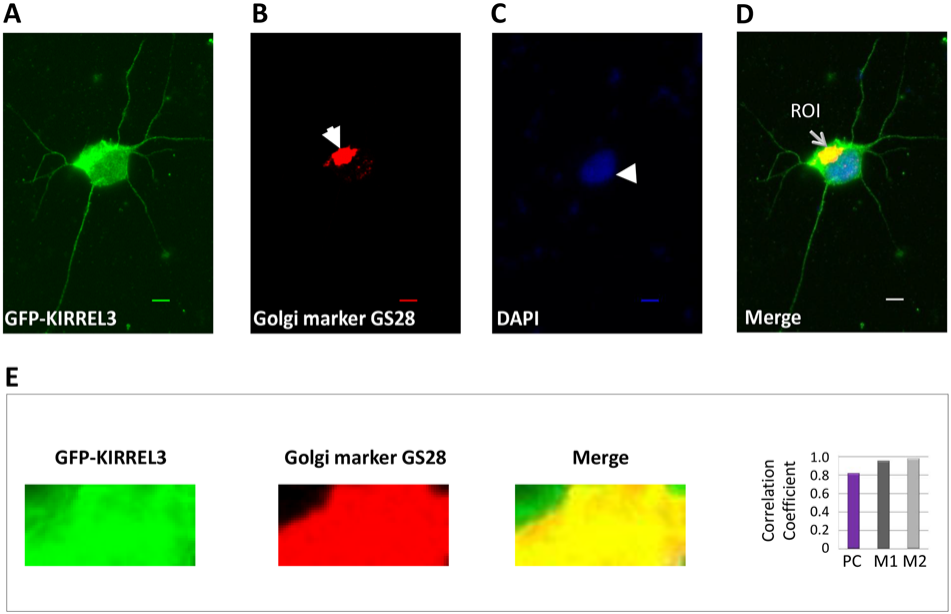
KIRREL3 localizes to the Golgi complex. Rat PNCs overexpressing the GFP-tagged KIRREL3 were immunostained with GFP antibody (green signal) (A) and the Golgi marker GS28 antibody (red signal, solid arrow) (B). Nuclei were stained with DAPI (blue signal, arrow head) (C). A distinct localized yellow signal (thin arrow) in the merged image (D) suggested the colocalization of KIRREL3 with the Golgi apparatus. Enlarged overlay images and individual red and green channels for a region of interest (ROI) are shown (E). The degree of overlap between the green and red signals was statistically analyzed (E) and expressed with Pearson’s correlation coefficient (PC) and Mander’s colocalization coefficients (M1 and M2). Bar, 20μm.

KIRREL3-specifc signals and colocalization signals were observed in the perinuclear region (Fig 4A and 4B). This localization of KIRREL3 resembled the characteristic morphology of the Golgi complex. Thus, we further evaluated the subcellular localization of KIRREL3 by immunofluorescence experiments. GFP tagged KIRREL3 was transfected into rat PNCs and the double labeling experiments with GFP antibody and GS28 antibody, a Golgi apparatus marker, were performed. KIRREL3-specific intense green fluorescence was detected in the perinuclear region together with red fluorescence of GS28 indicating the subcellular Golgi localization of KIRREL3 (Fig 6D). Quantitative colocalization analysis calculated high values for the Pearson’s correlation coefficient and Mander’s colocalization coefficients within the region of interest, which strongly confirmed the subcellular Golgi localization of KIRREL3 (Fig 6E and Table 2).

**Fig 7:**
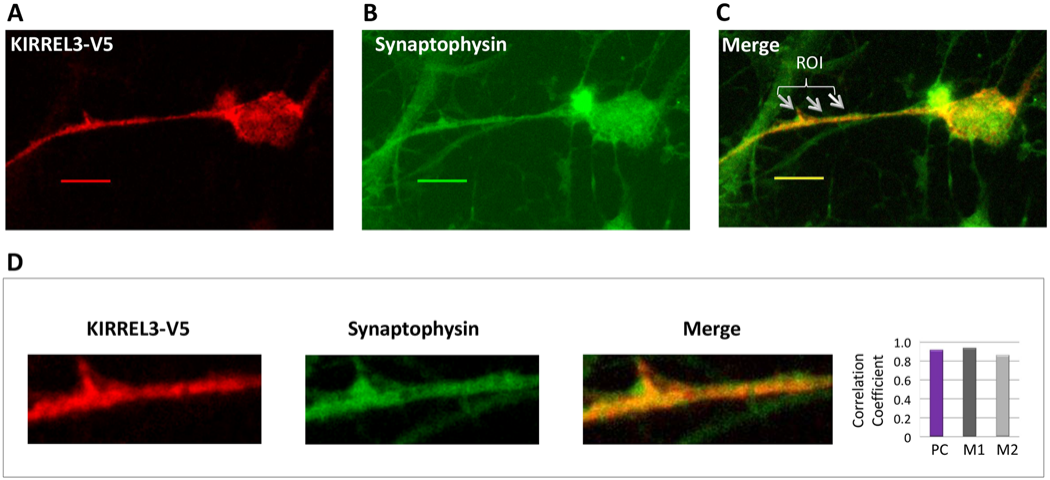
KIRREL3 colocalizes with the synaptic vesicles marker synaptophysin. Rat PNCs overexpressing KIRREL3-V5 (red signal) (A) were immunostained with synaptophysin (green signal) (B). Yellow/orange signals (arrows in merged image) (C) in selected areas in a cell with KIRREL3-V5 suggested the colocalization of KIRREL3 with the synaptic vesicles. An enlarged overlay image and individual red and green channels for an area with ROI are shown (D). The degree of colocalization between the red and green signals was statistically analyzed (D) and expressed with Pearson’s correlation coefficient (PC) and Mander’s colocalization coefficients (M1 and M2). Bar, 20μm.

Furthermore, a punctate distribution of KIRREL3 in cell bodies and neurite processes were also observed (Fig 4A and 4B). This punctuate appearance is indicative of potential localization of KIRREL3 to synaptic/secretary vesicles. Thus, we used double staining with V5 antibody and synpatophysin antibody, an integral synaptic vesicles membrane protein marker, and confirmed that KIRREL3 was cololcalized with synpatophysin in rat PNCs (Fig 7). Quantitative colocalization analysis also confirmed the synaptic vesicle localization of KIRREL3 in selected regions (Fig 7D and Table 2).

### Deletions of *MYO16* and *MAP1B* in patients with neurodevelopmental disorders

Among unrelated patients referred for clinical diagnostic testing who were found to have CNVs, we identified two patients with neurodevelopmental disorders and deletions encompassing the *MYO16* gene and one patient with a deletion encompassing the *MAP1B* gene (Table 3).

**Table 3:** Summary of CNVs involving KIRREL3 interacting proteins MYO16, MAP1B, and ATP1B1 in patients with neurodevelopmental disorders.

Patient ID and Phenotype	CNV	Chromosomal Location*	Interval	OMIM Genes	Reference
DGDP067A Receptive-expressive language disorder, microcephaly, visual impairment, astigmatism, strabismus, torticollis, significant delay in cognitive and motor development	DEL, ring chr	**13q33.3** n/a—n/a	~10.7 Mb	25 genes including ***MYO16***	Present study
	DEL, inherited from normal mother	**7q36.3** n/a—n/a	717 kb	-	Present study
10–12641 History of developmental delay, ADHD, autism, microcephaly, congenital grouped pigmentation of the retinal pigment epithelium, abnormal white matter on cranial MRI	DEL	**13q33.3q34** 106,919,677–109,954,934	3.04 Mb	6 genes including ***MYO16***	Present study
	DUP	**12q24.31** 123,522,421–123,785,941	264 kb	*NCOR2*	Present study
	DEL	**11p15.4** 6,739,589–6,850,118	111 kb	-	Present study
10–17471 Intellectual disability, seizures, borderline microcephaly, normal cranial MRI	DEL *de novo*	**5q13.2** 71,221,928–72,188,118	966 kb	3 genes including ***MAP1B***	Present study
	DEL *de novo*	**7q21.11** 79,661,304–84,240,429	4.6 Mb	8 genes	Present study
1056/9897 Moderate intellectual disability, upslanting palpebral fissures, retrognathia	DEL	**chr13:** 107,190,506–109,368,996	2.09 Mb	6 genes including ***MYO16***	[8–9]
DECIPHER 280488 Global developmental delay	DEL	**chr13:** 106,884,343–110,711,191	3.83 Mb	8 genes including ***MYO16***	[10–11]
DECIPHER 280995 Intellectual disability, moderate behavioral/psychiatric abnormality, seizures, constitutive in mother; Mother affected with related or similar phenotype	DEL, maternal	**chr13**: 102,864,674–109,548,530	6.68 Mb	17 genes including ***MYO16***	[10–11]
DECIPHER 2365 Intellectual disability, coarse facial features, self-mutilation	DEL	**chr1:** 166,047,706–171,783,874	5.74 Mb	47 genes including ***ATP1B1***	[10–11]
DECIPHER 253856 Intellectual disability, abnormality of the face, horseshoe kidney, malformation of the heart and great vessels	DEL	**chr1:** 164,501,003–171,424,595	6.92 Mb	51 genes including ***ATP1B1***	[10–11]
DECIPHER 285697 Intellectual disability, coarse facial features, self-mutilation	DEL	**chr1:** 167,238,254–171,783,874	5.74 Mb	47 genes including ***ATP1B1***	[10–11]

*NCBI Build 36

Patient DGDP067A (Table 3) with ID, developmental disorder (DD) and two deletions has a ring chromosome 13 with a 10.7 Mb deletion from 13q33.3 to the telomeric end. This *de novo* deletion, detected by Agilent 105K oligonucleotide array, contains several genes including *MYO16* at 13q33.3. The deletion was confirmed by FISH analysis with a BAC clone RP11-789H4. The second deletion, del(7)(q36.3), approximately 717 kb in size, is inherited from her healthy mother and thus most likely a polymorphism. The proband has mixed receptive-expressive language developmental disorder, microcephaly, visual impairment, astigmatism, strabismus, torticollis, and significant delays in speech, cognitive and motor development. At 4 years of age she is mentally about 18 to 20 months old.

Patient 10–12641 (Table 3) is a 10 yr 6 month old male with a history of development delay, ADHD, autism, congenital grouped pigmentation of the retinal pigmentation epithelium, abnormal white matter on cranial MRI, microcephaly, and three chromosome changes on microarray analysis (Affymetrix Genome-wide SNP 6.0 microarray system). A copy number loss of approximately 3 Mb at 13q33.3q34 included 6 genes including the *MYO16* gene. In addition, a copy number gain of approximately 264 kb on chromosome 12q24.31 and a copy number loss of 111 kb on chromosome 11p15.4 were also noted. Quantitative PCR (qPCR) analysis confirmed all three chromosomal changes.

Patient 10–17471 (Table 3) is a 17 yr old female with ID, seizures, microcephaly and normal cranial MRI. The patient showed a copy number loss of approximately 966 kb on chromosome 5q13.2 including the *MAP1B* gene and a second copy number loss of approximately 4.6 Mb on chromosome 7q21.11. Both deletions were confirmed using qPCR analysis.

In addition, deletions of *MYO16* and *ATP1B* genes have been also noted in additional unrelated patients with neurodevelopmental disorders (NDDs) in other published studies (Table 3) ([8–10] and see acknowledgments for details). Recently, a patient with mild ID, upslanting palpebral fissures, retroganthia and a *de novo* 2.1 Mb microdeletion encompassing the *MYO16* gene has been reported (Patient 1056/9897 in Table 3) [8–9]. In addition, the DECIPHER database [10–11] lists a 3.83 Mb copy number loss (chr13: 106,884,343–110,711,191; NCBI Build 36) including the *MYO16* gene in a male patient with global developmental delay and an apparently pathogenic maternally inherited 6.68 Mb CNV (chr13: 102,864,674–109,548,530; NCBI Build 36) in a female patient with ID, seizures and moderate behavioral/psychiatric abnormality (Table 3). The patient’s mother also reportedly has related or similar phenotype. The telomeric deletion boundary in this patient is predicted to disrupt the *MYO16* gene. Furthermore, a genome wide association study suggested a potential autism association signal at 13q13.3 near *MYO16* [12]. Three unrelated patients in the DECIPHER database with deletions involving the *ATP1B1* gene shared ID as a common clinical phenotype (Table 3). No CNVs including either *MYO16*, *MAP1B*, or the *ATP1B1* gene were listed in genome variant in human genome database [13] suggesting a likely clinical significance of CNVs noted here in patients with NDDs.

## Discussion

The *KIRREL3* gene, a mammalian homologue of the gene *kirre* (kin of irregular chiasm C-roughest) of *Drosophila melanogaster*, encodes a putative synaptic adhesion protein of the immunoglobulin superfamily. KIRREL3 contains five Ig like domains in its extracellular portion and a PDZ domain-binding motif in its cytoplasmic portion. Expression of the *KIRREL3* gene was detected exclusively in human fetal and adult brain [1]. Consistent with a vital role in brain development and function, spatiotemporal expression patterns of the mouse gene, *mKirre*, in developing and adult brain regions, including postnatal hippocampus, suggest a role for *mKirre* in axonal projections and synapse formation [14]. We have recently identified a potential role for human KIRREL3 in neurodevelopment [1]. Subsequently, several independent studies also linked defects in KIRREL3 to neurological and cognitive disorders [2–5]. To help define molecular mechanisms underlying the physiological action of KIRREL3 and its role in cognitive function, it is important to establish how KIRREL3 mediates its physiological functions in neurons. Thus we sought to identify proteins that are involved in this process through their interactions with KIRREL3. Using the yeast two-hybrid screen, we identified two brain expressed proteins, MAP1BLC1 and MYO16, that interact with KIRREL3-ECD. Three proteins, ATP1B1, UFC1, and SHMT2, were found to be associated with KIRREL3-ICD (Table 1). All the interactions were confirmed by co-immunoprecipitation and colocalization experiments. Furthermore, we demonstrated perinuclear localization and punctuate distribution of KIRREL3 in primary neuronal cells by immunocytochemical analysis indicating its localization to the Golgi complex and the synaptic secretary vesicles, suggesting a likely role for KIRREL3 in vesicular transport system of neuronal cells.

KIRREL3-ICD interacting protein ATP1B1 belongs to the family of Na^+^/K^+^ and H^+^/K^+^ ATPases beta chain proteins, and to the subfamily of Na^+^/K^+^-ATPases. Recently, ATP1B1 has been found to be regulated by neuron-specific transcription factor Sp4 and shown to play an important role in mediating the tight coupling between energy production, neuronal activity and energy consumption [15]. UFC1 is one of the enzymes involved in modification of proteins with the ubiquitin-like molecule ubiquitin-fold modifier-1 (Ufm1). UFC1 interacts with cytoplasmic domain of a cell adhesion molecule, NCAM140, and results in its increased endocytosis [16]. Recently, NCAM140/NCAM120-mediated Fyn activation has been shown to promote GABAergic synapse maturation in postnatal cortex [17]. NCAMs interact with several cytoskeleton proteins and signaling molecules involved in synaptic plasticity and many neurological disorders [18]. SHMT2 is the mitochondrial form of a pyridoxal phosphate-dependent enzyme that catalyzes the reversible reaction of serine and tetrahydrofolate to glycine and 5,10-methylene tetrahydrofolate and recently shown to be a potential cancer driver gene [19].

KIRREL3-ECD interacting proteins, MAP1B and MYO16, provide further clues to its cellular function and a likely involvement of KIRREL3 in regulation of the synaptic actin cytoskeleton. Finding of KIRREL3-ECD interactions with cytoplasmic proteins are not unusual as KIRREL3 localization has been noted in cytoplasm [1]. Myosins are actin-based motor molecules with ATPase activity. A recent study showed that assembly of the F-actin network at synapse requires a direct interaction between the cell adhesion molecule, *SYG-1*, a *C*.*elegans* ortholog of human KIRREL3, and a key regulator of actin cytoskeleton, the WVE-1/WAVE regulatory complex (WRC) [20]. Interestingly, MYO16 (NYAP3) has recently been identified as a novel regulator of PI3K in neurons and links PI3K signaling to WAVE1 signaling in neurons. Furthermore, MYO16 cosediments with F-actin in an ATP-sensitive manner [21]. MYO16 is expressed predominantly in developing neurons and present throughout the somal cytoplasm as well as along the entire length of all neurite processes ([21] and present study). It activates PI3K and concomitantly recruits the WAVE1 complex to the close vicinity of PI3K and regulates neuronal morphogenesis [22]. Interestingly, KIRREL3 also showed signals in somal cytoplasm and in punctuate structure along neurite processes.

MAP1B is a classical microtubule-associated cytoskeleton protein that consists of heavy chain and light chain (LC) and plays important roles in the regulation of neuronal morphogenesis. MAP1BLC1 is also known to interact with diverse ionotropic receptors at the postsynapse. *MAP1B* deficiency is shown to be accompanied by abnormal actin microfilament polymerization and dramatic changes in the activity of small GTPases controlling the actin cytoskeleton [23]. Mice deficient in *Map1b* showed impaired long-term potentiation [24] and also a distinct behavioral phenotype and altered retinal function [25].

Cell-adhesion molecules of the immunoglobulin superfamily play critical roles in brain development, as well as maintaining synaptic structure, function and plasticity. Growing evidence suggests that ID, autism and other neurocognitive developmental disorders might be caused by defects in synapse structure and function [26]. Several of these KIRREL3 interacting proteins have previously been linked to neurological and cognitive disorders. Previously, we and others have shown that KIRREL3 cytoplasmic domain interacts with CASK, a synaptic scaffolding protein, in neuronal cells [1, 7]. CASK localizes to synaptic active zone and binds to presynaptic β-neurexin and calcium channels [27]. The deletion of *Cask* in mice impairs synaptic function [28], and defects of the human *CASK* gene cause X-linked ID [29–31]. Recently, an ATP1B1 nonsense mutation, p.R143X, was identified in a patient with autistic features [32]. In addition, several deletions that included the *ATP1B1* gene were reported in patients with ID/global developmental delay (Table 3) [10]. Genetic association has been found between the *MYO16* gene and autism in two large cohorts (AGRE and ACC) of European ancestry and replicated in two other cohorts (CAP and CART). Recently, we and others identified deletions of *MYO16* in patients with ID, which further suggests its role in neurodevelopment [8–10]. Previous study with the yeast two-hybrid system revealed that *MYO16* associated with neurexin 1 (NRXN1) [33]. Neurexins are neuronal cell surface protein and mutations of *NRXN1* have been reported in patients with ID and autism spectrum disorder [34–35]. MAP1B has been associated with human neurological disorders, such as giant axonal neuropathy, fragile-X syndrome, and spinocerebellar ataxia type 1 [36–38].

We speculate KIRREL3 interacting proteins to be potential candidates for NDDs such as ID and ASDs. Findings of deletions or defects in several of the KIRREL3-interacting proteins in patients with NDDs further strengthen this notion and indicate a suggestive clinical association. Thus extensive additional analyses are warranted to understand these clinical and potential pathogenic associations. Altogether, our study provides valuable information for understanding KIRREL3 involvement in cognitive and behavioral disorders and provides further insights into molecular mechanisms of its action, in particular its potential involvement in synaptic actin-cytoskeleton through MYO16-WAVE1 complex. The latter function is considered critical as the ECD of SYG-1 alone is capable of rescuing the terminal phenotype of the *syg-1* mutant [39]. Establishment of connection with MAP1B and MYO16 opens avenues for examining KIRREL3 physiological function in detail and the importance of F-actin mediated regulation of synaptic cytoskeleton in neurocognitive developmental disorders.

## Materials and Methods

### Human subjects and CNV analysis

The study protocol, consent form and privacy practices were reviewed and approved by the Institutional Review Board of Self Regional Healthcare. Patients described in this study were referred to clinical diagnostic testing with their informed written parental consent. Copy number variation analysis, qPCR, and FISH were performed as previously described [40].

### Molecular cloning of bait constructs for yeast two-hybrid screening

The intracellular domain and extracellular domain of KIRREL3 (KIRREL3-ICD and KIRREL3-ECD, respectively) were constructed into the bait vector pGBKT7 (containing the *GAL4* DNA binding domain) using the In-Fusion Dry-Down PCR Cloning Kit (Clontech). The pGBKT7-KIRREL3-ICD and pGBKT7-KIRREL3-ECD constructs were then transformed into Top10 competent *E*. *coli* cells following the manufacturer’s protocol and purified using a QIAprep Spin Miniprep Kit (Qiagen). The two constructs were confirmed by sequencing with insert-specific forward primers and vector-specific reverse primers.

### Auto-activation test

To test for auto-activation, *S*. *cerevisiae* strain AH109 was transformed with pGBKT7-KIRREL3-ICD and pGBKT7-KIRREL3-ECD using the small-scale transformation protocol as described in the Matchmaker *GAL4* Two-Hybrid System 3 User Manual (Clontech). Cells containing pGBKT7-KIRREL3-ICD or pGBKT7-KIRREL3-ECD were then spread on SD/-Trp, SD/-Trp/X-α-Gal, SD/-Leu/-Trp, SD/-His/-Leu/-Trp, and SD/-Ade/-His/-Leu/-Trp /X-α-Gal agar plates (QDO/X-α-Gal plates). White colonies were observed on SD/-Trp and SD/-Trp/X-α-Gal plates but not on other plates.

### Yeast two-hybrid screening

The bait constructs, pGBKT7-KIRREL3-ICD and pGBKT7-KIRREL3-ECD, were transformed into AH109 yeast strain. The human fetal brain cDNA library (Clontech), which was engineered in the prey vector pGADT7, was transformed in the yeast strain Y187. AH109 strains harboring pGBKT7 plasmids were maintained in minimal SD media with tryptophan dropout supplement (SD/-Trp), while Y187 strains harboring pGADT7 plasmids were maintained in minimal SD media with leucine dropout supplement (SD/-Leu). Yeast mating was carried out as described in the Matchmaker Gold Yeast Two-Hybrid System User Manual. Five to seven days after plating the mated diploid yeasts, the QDO/X-α-Gal plates were scored for positive protein-protein interactions. QDO/X-α-Gal plates represent the high selection stringency for eliminating possible false positives. The mating between AH109 transformed with a plasmid expressing the GAL4 binding domain (BD)-p53 fusion protein and Y187 transformed with a plasmid expressing the GAL4 activation domain (AD)-T (SV40 T antigen) fusion protein was used as the positive control. The mating between AH109 transformed with a plasmid expressing the BD-Lamin fusion protein and Y187 transformed with a plasmid expressing AD-T was used as the negative control. Growth of the diploid yeast cells representing the positive interactions was further analyzed. The positive AD-cDNA clones were characterized by DNA sequencing and these DNA sequences were analyzed by BLAST search for GenBank database.

### Confirmation of positive interactions by yeast mating experiment

To confirm the positive interactions in yeast, candidate genes were engineered into the pGADT7 vector using the In-Fusion Dry-Down PCR Cloning Kit (Clontech). The constructs were confirmed by sequencing with insert-specific primers. The bait and prey plasmids were transformed into AH109 and Y187 cells, respectively. Y2H experiments were performed following the standard procedure as described in the Matchmaker Gold Yeast Two-Hybrid System User Manual (Clontech). All the potential positive interactions generated blue or light blue colonies on SD/-Ade/-His/-Leu/-Trp /X-α-Gal agar plates.

### Molecular cloning of expression constructs for immunofluorescence and coimmunoprecipitation studies

MAP1BLC1, ATP1B1, UFC1, and SHMT2 were constructed in the N-terminal p3XFLAG-CMV vector (Invitrogen), which tagged with N-terminal FLAG, using In-Fusion Dry-Down PCR Cloning Kit (Clontech). MYO16 was engineered in the pEGFP-C3 vector (Clontech), which tagged with N-terminal GFP, using In-Fusion Dry-Down PCR Cloning Kit (Clontech). Full-length KIRREL3, KIRREL3-ECD, and KIRREL3-ICD were subcloned into pcDNA3.1/NT-GFP-TOPO, which tagged with N-terminal GFP, with GFP Fusion TOPO TA Expression Kits (Invitrogen). KIRREL3-ECD and KIRREL3-ICD were subcloned into pcDNA3.1D/V5-His-TOPO vector, which tagged with C-terminal V5, with In-Fusion Dry-Down PCR Cloning Kit. The construction of V5-tagged KIRREL3 was described previously [1].

### Neuronal cell culture and transfection

Human embryonic kidney cells (HEK293H; Invitrogen, Life Sciences) were maintained in Dulbecco’s minimal essential medium (DMEM, sigma) supplemented with 10% fetal bovine serum (FBS, Atlanta Biologicals, Norcross, GA), L-glutamine (Sigma), and penicillin/streptomycin (Sigma). Primary rat neuronal cells were derived from live neuronal tissues, which were isolated from micro-surgically dissected embryos (E17-18) and the cortical hemispheres of Sprague Dawley rats. The cells were maintained in neurobasal media (Invitrogen) with 2% B-27 serum-free supplement (Invitrogen), L-glutamine, penicillin/streptomycin, 20 ng/ml *epidermal growth factor* (EGF, Millipore), and 20 ng/ml *fibroblast growth factor* (FGF, Millipore). After cells differentiation, 20 μM amino-phosphonovalerate acid (APV, Ascent Scientific) was added to the growth media. All the cell lines were maintained at 37°C and 5% CO2. PNC cells were transfected with lipofectamine ltx with Plus (Invitrogen) and all the other cells were transfected with lipofectamine 2000 (Invitrogen) using conditions recommended by the supplier. During transfection, cells were maintained in serum and antibiotic-free medium. Following at least 5hs of exposure to DNA-lipofectamine mixture, cells were refed with medium. All animals were maintained in the animal facility of Lander University. All of experimental protocols were approved by the Institutional Animal Care and Use Committee of Lander University, Greenwood, SC.

### Antibodies and indirect immunofluorescence staining

Cells grown on glass coverslips were fixed and permeabilized with 4% paraformaldehyde (Sigma) and 0.1% Triton X-100 (ICN Biomedicals) in PBS. Fixed cells were blocked in blocking buffer (2% horse serum and 0.4% BSA in PBS) for 30 minutes. Cells were then incubated with primary antibodies diluted in blocking buffer at appropriate concentrations for one hour. The primary antibodies were added simultaneously for the double staining. For the study of V5-tagged KIRREL3 and GFP-tagged MYO16, the mouse anti-V5 antibody (Invitrogen) was used at a 1:5000 dilution. Given the strong endogenous signal of GFP tag, no primary antibody was applied on GFP-tagged MYO16. For the study of V5-tagged KIRREL3 and FLAG-tagged MAP1BLC1/ATP1B1/UFC1/SHMT2, the mouse anti-V5 antibody and rabbit anti-FLAG antibody were used at 1:5000 and 1:800 dilutions, respectively. For the KIRREL3 Golgi localization study, mouse anti-GS28 antibody (BD Biosciences) was used at 1: 200 dilution. For the synaptic vesicle studies, goat anti-synaptophysin antibody (Santa Cruz Biotechnology) was used at 1:200 dilution. Subsequent antibody detection was carried out using Alexa Fluor 488 Chicken Anti*-*goat IgG (Molecular Probes), Alexa Fluor 488 Chicken Anti*-*rabbit IgG (Molecular Probes), and Alexa Fluor 594 Chicken Anti*-*Mouse IgG (Molecular Probes). Nuclear staining was performed using 4'-6-Diamidino-2-phenylindole (DAPI) (Molecular Probes). Slides were then viewed under the Zeiss AxioVision A10 observer-A1 microscope. Images were captured using a 63 X oil objective lens.

Colocalization analysis was performed using JACoP plugin embedded in the visualization and analysis software ImageJ version1.45s [41–42]. Analysis was performed on region(s) of interest (ROI) selected for each dye. ROIs were defined around the regions with KIRREL3 signal (red signal: Figs 4, 5 and 7; green signal: Fig 6) and with pair-wised green signal (Fig 4: MAP1BLC1 or MYO16; Fig 5: ATP1B1, UFC1, or SHMT2; Fig 7: Synaptophysin) or pair-wised red signal (Fig 6: Golgi). Each colored image was split into respective red, green, and blue channels. Enlarged overlay images and individual channels for each ROI are presented in respective figures. Using Costes' method of automatic thresholding, a Pearson's coefficient (PC) was calculated for pixels within all of the calculated regions of interest in an image where Alexa 488 and Alexa 594 fluorescence were each detected at levels significantly above background. Mander’s coefficients, M1 (representing the fraction of red signal overlapped with green signal) and M2 (representing the fraction of green signal overlapped with red signal), were calculated to determine the degree of overlap between the corresponding regions of detected signals at default settings. A total number of 50–75 cells from at least 3 individual experiments were studied and 10–15 cells were selected for further evaluation. For each cell, one or two ROIs (a total number of n = 15–30 ROIs) were quantitatively analyzed.

Negative control for colocalization analysis included KIRREL3-V5 in combination with either GFP-empty vector or BAP-FLAG negative control. Similarly, an empty GFP vector without KIRREL3 for Golgi or a LacZ-V5 negative control for Synaptophysin colocalization studies were included as negative control.

### Co-immunoprecipitation and western blot analysis

HEK293H cells grown in 60 mm-diameter tissue culture plates were transiently transfected with various expression plasmids including 3μg of V5-tagged KIRREL3 or KIRREL3 variants and 3μg of GFP-tagged MYO16; 3μg of GFP-tagged KIRREL3 or KIRREL3 variants and 3μg of FLAG-tagged MAP1BLC1/ ATP1B1/ UFC1/ SHMT2 expression plasmids using Lipofectamine 2000 (Life Technologies, Inc.) under conditions specified by the supplier. 24 hours after transfection, cells were washed 1X with ice-cold PBS and incubated with 1% NP-40 lysis buffer (1% NP-40; 150 mM NaCl; 50 mM Tris, pH8.0) plus protease inhibitor cocktail (Sigma) for at least 10 minutes on ice. Proteins were sonicated and the extracts were then collected by 10 minutes centrifuge at 10,000Xg at 4°C. Cleared supernatant were quantified by Bradford and incubated with anti-rabbit antibody conjugated magnetic beads (Pierce Inc.), which were coated with 1% BSA in 1% NP40 lysis buffer with protease inhibitor cocktail, on a spinning rotator at 4°C for 2hrs. Precleared supernatant from each experimental group was incubated with 1μg anti-V5 antibody (anti-rabbit, sigma) or 1μg anti-GFP antibody (anti-rabbit, Immunology Consultant Laboratory, Inc.) on a spinning rotator at 4°C for 2hrs. Coated magnetic beads were added to the lysate-antibody mixture and incubated on the rotator at 4°C overnight. The immunoprecipitation reactions were washed three times with 1% NP40 lysis buffer with 1% BSA for 15min each at 4°C and once with 1% NP40 lysis buffer for 20min at 4°C. Bound protein was eluted with 40μl 1X sodium dodecyl sulfate (SDS) sample buffer and, following SDS-PAGE, subjected to western blot analysis to detect V5-tagged proteins, GFP-tagged proteins, FLAG-tagged proteins, or endogenous MAP1BLC1 protein. For the study of V5 tagged KIRREL3 or variants and GFP tagged MYO16 the mouse anti-V5 antibody (Invitrogen) at 1:5000 and the mouse anti-GFP antibody (Roche) at 1:2000 were used. For the study of GFP tagged KIRREL3 or variants and FLAG tagged MAP1BLC1/ ATP1B1/ UFC1/ SHMT2, the mouse anti-GFP antibody (Roche) at 1:2000 and the mouse anti-FLAG antibody (Sigma) at 1:4000 and were used. Mouse anti-MAP1BCL1 antibody (Santa Cruz Biotechnology, Inc.) was used at 1:200 dilution. Immunoreactive bands were detected by SuperSignal West Dura (Pierce Inc.) with standard x-ray film (Kodak, Rochester, NY).
